# Cellulose Sulfate Nanofibers for Enhanced Ammonium Removal

**DOI:** 10.3390/nano14060507

**Published:** 2024-03-12

**Authors:** Ken I. Johnson, William Borges, Priyanka R. Sharma, Sunil K. Sharma, Hao-Yen Chang, Mortaga M. Abou-Krisha, Abdulrahman G. Alhamzani, Benjamin S. Hsiao

**Affiliations:** 1 Department of Chemistry, Stony Brook University, Stony Brook, New York, NY 11790, USApriyanka.sharma@wmich.edu (P.R.S.);; 2 Department of Biochemistry and Molecular Biology, Brown University, Providence, RI 02906, USA; 3 Department of Chemical and Paper Engineering, Western Michigan University, Kalamazoo, MI 49008, USA; 4 Department of Chemistry, College of Science, Imam Mohammad Ibn Saud Islamic University (IMSIU), Riyadh 11623, Saudi Arabia; mmaboukrisha@imamu.edu.sa (M.M.A.-K.); agalhamzani@imamu.edu.sa (A.G.A.)

**Keywords:** nanocellulose, ammonium removal, cellulose sulfate, jute

## Abstract

In this study, a sulfonation approach using chlorosulfonic acid (CSA) to prepare cellulose sulfate nanofibers (CSNFs) from raw jute fibers is demonstrated. Both elemental sulfur content and zeta potential in the CSNFs are found to increase with increasing CSA content used. However, the corresponding crystallinity in the CSNFs decreases with the increasing amount of CSA used due to degradation of cellulose chains under harsh acidic conditions. The ammonium adsorption results from the CSNFs with varying degrees of sulfonation were analyzed using the Langmuir isotherm model, and the analysis showed a very high maximum ammonium adsorption capacity (41.1 mg/g) under neutral pH, comparable to the best value from a synthetic hydrogel in the literature. The high ammonium adsorption capacity of the CSNFs was found to be maintained in a broad acidic range (pH = 2.5 to 6.5).

## 1. Introduction

Recent research activities in developing new sustainable materials from natural resources for water purification have gained notable interest beyond the subjects of biochar- and zeolite-based materials. Specifically, nanomaterials based on carboxylated cellulose nanofibers have attracted a great deal of attention due to several newly developed processes that can effectively oxidize the cellulose surface (converting the hydroxyl group into a carboxyl group on the C6 position of the glucose repeating unit), bring negative charges on the cellulose surface, facilitate the defibrillation process, and produce no waste [1,2]. However, there is one limitation of using nanocellulose with carboxylic acid or carboxylate functional groups, that is, its efficacy at different pH values. 

Similar limitations have also been noted in ion-exchanging materials containing carboxyl or carboxylate groups, which are termed weak acid cation exchangers (WACEs) [3]. In WACEs, the carboxylic acid (carboxyl) groups can be protonated, and the carboxylate groups are present in the form of a salt with a counter ion such as sodium. The hydrogen in the carboxyl group of WACEs cannot be exchanged by neutral salts, such as sodium chloride. Instead, they are highly reactive towards alkaline species, such as sodium hydroxide and calcium bicarbonate [4]. The hardness of water can be used to determine whether water has a relatively high concentration of dissolved multivalent cations (e.g., calcium and magnesium). If the alkalinity is greater than the hardness, the ions contributing to the hardness are removed (some sodium ions are also removed). The amount of ions (sodium and hardness) removed will be equal to the alkalinity neutralized via the carboxylic acid groups. If the hardness is greater than the alkalinity, only ions contributing to the hardness are removed. All ion-exchange systems have a degree of selectivity, and typically have greater affinity for ions with higher molecular weights and higher ionic charges [5]. A distinct exception to this trend exists with WACEs. The carboxylate ion, being a conjugate base and also an alkaline salt, reacts with acid to form carboxylic acid, and has a higher affinity for hydrogen than any other cation [6]. This is also observed in the regeneration processes of WACEs, which require only a dilute and stoichiometric solution of acid [7]. The implications of this property mean that bound impurities may be easily lost due to the interaction with acid, but also that the regeneration of WACEs is particularly efficient and cost-effective.

The hydrogen WACEs can be converted to an ionic WACE using alkaline salts, such as sodium bicarbonate [8]. Ionic WACEs can be used to exchange both alkaline and neutral salts, and generally follow the selectivity preference for ions with higher molecular weights and higher ionic charges. As a result, ionic WACEs cannot be regenerated using concentrated sodium chloride, but can be regenerated to the hydrogen WACE using a dilute and stoichiometric solution of acid, and subsequently converted to an ionic WACE using an alkaline salt. 

Strong acid cation-exchange (SACE) materials usually have a sulfonic acid functional group and follow the selectivity for ions with higher molecular weights and higher ionic charges. SACEs can be regenerated using concentrated sodium chloride, unlike WACEs, which must first be converted into a hydrogen WACE using an acid and then to an ionic WACE using a base [8]. This gives SACEs an advantage in that they require one less step in the regeneration process. Sulfur, being more electronegative than carbon, gains relatively more electron density from oxygen and takes the electron density away from hydrogen. SACEs act as strong acids; their affinity for hydrogen is lower than other monovalent cations, such as sodium and potassium. Compared with WACEs, SACEs have the advantage of operating in a broader pH range, faster kinetics, and a lack of swelling behaviors due to the relatively higher solubility of WACEs. 

In this study, cellulose sulfate nanofibers (CSNFs) were prepared from raw jute feedstock using a heterogenous sulfonation approach, involving the use of chlorosulfonic acid (CSA). This chemical process has been reported in the literature but has not been conducted on raw lignocellulosic biomass [9,10,11,12,13]. The structure, morphology, and chemical modification of the resulting CSNFs prepared using different CSA amounts were characterized by transmission electron microscopy (TEM), Fourier transform infrared spectroscopy (FTIR), nuclear magnetic resonance (NMR), wide-angle X-ray diffraction (WAXD), thermogravimetric analysis (TGA), elemental sulfur content, and zeta potential. The ammonium adsorption efficiency of the CSNFs in the suspension state was also tested. The results were analyzed using the Langmuir isotherm model to yield the maximum adsorption capacity, and the adsorption performance was evaluated at various pH conditions. 

## 2. Materials and Methods

### 2.1. Materials

Jute fibers were obtained from Toptrans Bangladesh Ltd. in Dhaka, Bangladesh. Anhydrous N,N-dimethylformamide (DMF) was purchased from Fisher Scientific, Waltham, MA, USA. Chlorosulfonic acid (CSA, 99%) was purchased from VWR, Radnor, PA, USA. Ammonium chloride (98%) and hydrochloric acid (1.0 N) were purchased from Sigma Aldrich, St. Louis, MO, USA. Sodium hydroxide (99%) was purchased from Macron Fine Chemicals, Center Valley, PA, USA. All chemicals were used without further purification. Cellulose dialysis tubing with a molecular weight cutoff of 12–14 kDa made by Spectra/Por was also purchased from VWR, Radnor, PA, USA. 

### 2.2. Preparation of Cellulose Sulfate Nanofibers

Reaction conditions for preparation of cellulose sulfate nanofibers (CSNFs) are given in Table 1. In all reactions, 2.0 g of ground raw jute was suspended in 35 mL of anhydrous DMF for 1 h. An appropriate amount of CSA ranging from 1.0 to 2.5 mL was added (by dropwise addition) to the suspension using a serological pipet. The suspension was then stirred at room temperature for 6 h. The reaction was then quenched by adding 100 mL of deionized (DI) water, and allowed to stir for 30 min. The resulting suspension was vacuum-filtered until the fibers began to swell. Once the fibers swelled to a certain point, vacuum filtration became inefficient, at which point the sample was suspended in a dialysis tube. The dialysis water was changed twice a day until the conductivity of the wash water did not change over a 24 h period. 

After dialysis, samples were placed in 500 mL media bottles and diluted to 500 mL using DI water. The suspensions were passed through a high-pressure homogenizer at 400 bars, which noticeably changed the viscosity to a viscous but flowable liquid. After letting the suspension equilibrate over 24 h, a small portion was weighed and dried to determine the weight percent (wt%) and product yield of the samples. 

### 2.3. Cellulose Sulfate Nanofiber Characterization

#### 2.3.1. Transmission Electron Microscopy

Transmission electron microscopy (TEM) was conducted on a JEOL JEM 1400 instrument (Tokyo, Japan) at an accelerating voltage of 120 kV. The samples were prepared on 300-mesh copper grids (Ted Pella Inc., Redding, CA, USA) by casting 10 µL of a 0.01 wt% sample onto a grid. After removing the excess fluid, the sample was stained with 10 µL of 2 wt% aqueous uranyl acetate solution. The excess solution was subsequently removed, and the coated grid was left to air-dry.

#### 2.3.2. Fourier Transform Infrared Spectroscopy 

Fourier transform infrared spectroscopy (FTIR) was recorded on a Nicolet iS10 FT-IR Spectrometer by Thermo Scientific, Waltham, MA, USA, using the attenuated total reflectance (ATR) mode. Each spectrum was averaged from the measurements of 4 scans. The chosen instrument was equipped with a DTGS KBr detector, a KBr beam splitter, Smart iTR accessory, and a diamond window. Each sample was scanned from 400 to 4000 cm^−1^. 

#### 2.3.3. ^13^C Cross Polarization Magic-Angle Spinning Nuclear Magnetic Resonance

^13^C Cross polarization magic-angle spinning nuclear magnetic resonance (CPMAS-NMR) measurements of the chosen samples were carried out by a Bruker Utrashield 500 WB plus (500 MHz) instrumen (Bruker Corporation, Billerica, MA, USA), which was equipped with a 2.5 mm triple resonance MAS NMR probe, capable of spinning samples up to 35 KHz. The chosen resonance frequency was 10,000 Hz, and the samples were spun at the magic angle with a speed of 10 KHz. 

#### 2.3.4. Sulfur Elemental Analysis

A FlashEA 1112 instrument (Thermo Scientific, Waltham, MA, USA) was used to determine the elemental sulfur content of the samples. This instrument was equipped with a flash dynamic combustion chamber, capable of reaching a temperature of 1800 °C. A gas chromatography and thermal conductivity detector were used to quantify the sulfur content. 

#### 2.3.5. Wide-Angle X-ray Diffraction 

A MiniFlex instrument from Rigaku Japan was used to measure and record wide-angle X-ray diffraction (WAXD) patterns. The samples were measured from 5 degrees to 100 degrees (2 theta (2ϴ) diffraction angle), in steps of 0.02 degrees and at a rate of 5 degrees per minute. The scan axis was set to a theta/2-theta continuous mode, where the intensity measured was in counts per second. The voltage and current were set to 40 kV and 15 mA, respectively, using Cu Kα radiation. An incident-side and receiving-side soller slit of 5.0 degrees along with an incident beam divergence-limiting slit of 1.25 degrees were used in this measurement. 

The WAXD patterns were analyzed as follows. The measured patterns were first baselined using a straight line of constant value equal to that of the lowest value in the range of 5–45° 2ϴ. Six Gaussian curves were then used to fit the diffraction pattern, five for each lattice plane of cellulose crystals and one for the amorphous background. In the fitting procedure, the centers for 101, $10\bar{1}$, amorphous background, 021, 002, and 040 peaks were constrained to 14–16, 16–18, 19.5–22.5, 19.5–21.5, 22.5–23.5, and 34.5–35.5° 2ϴ, respectively. Constraints for the peak centers were disabled after several fitting iterations, so that constraints did not heavily influence the final peak positions. After several iterations, the full width at half maximum (FWHM) for the strongest crystalline peak, 002, was determined. This FWHM (002) was used to set the constraints for all other crystalline peaks in the pattern, specifically ± 0.4. In other words, the 002 peak in each sample was used as a reference to guide the FWHM determination for other crystalline peaks. In contrast, the FWHM for the amorphous cellulose (peak 3) was not constrained. The typical FWHM for amorphous cellulose was greater than 9° because in some instances the FWHM had to be initially set to a high value, such as 9, before fittings would converge. In general, the peak area was not constrained, except for constraining the area to positive values only. Fittings were iterated until converged, with a tolerance of $1.0\times 10^{-6}$. 

#### 2.3.6. Thermogravimetric Analysis 

Thermogravimetric analysis (TGA) was performed on a TA Q50 instrument (TA Instruments, New Castle, DE, USA). In this measurement, a sample was heated from 25 °C to 850 °C at 10 °C per minute, under a nitrogen atmosphere, where its mass was monitored over a temperature range. Sample was loaded and measured on a platinum pan. The derivative weight, shown as %/min, was also plotted against temperature to obtain derivative thermogravimetry (DTG) profiles, which showed more clearly the small changes in weight during the heating scan.

#### 2.3.7. Zeta Potential

The averaged zeta potential values of the samples were taken on a ZetaProbe Analyzer by Colloidal Dynamics, Ponte Vedra Beach, FL, USA. Samples were measured with the assumption of a dielectric constant of 5.0 and a density ratio of 1.5 g/mL. In this test, samples were stirred at 300 rpm and titrated with an auto-burette. DI water was used as a solvent for all samples. The instrument was equipped with a niobium electrokinetic sonic amplitude electrode and calibrated with a KSiW suspension. Samples were treated with either hydrochloric acid or sodium hydroxide using an auto-burette to achieve the desired pH before the analysis.

#### 2.3.8. Ammonium Ion-Selective Electrode 

Ammonium concentration was determined using an ammonium ion-selective electrode (ISE). The IntelliCAL ISENH4181 probe by Hach (Hach Company, Loveland, CO, USA) was equipped with a non-refillable Dritek gel reference and double junction encased in an epoxy body. An Ag/AgCl reference electrode and solid-state PVC membrane sensor were also included in this instrument.

#### 2.3.9. Ammonium Adsorption Data Analysis Using Langmuir Isotherm Model

The ammonium adsorption data analysis using the Langmuir isotherm model [14] was carried out as follows. The Langmuir isotherm model can be expressed as

(1) $$Langmuir\;equation\;Q_{e}=\frac{Q_{m}{bC}_{e}}{1+{bC}_{e}}$$

where *Q_e_* represents the equilibrium adsorption capacity, *C_e_* is the equilibrium concentration of ammonium, *Q_m_* is the monolayer maximum adsorption capacity, and *b* is the Langmuir adsorption constant. *Q_m_* can be determined as the inverse slope calculated from the linear fit when plotting *C_e_/Q_e_* against *C_e_*.

## 3. Results and Discussion

The objective of this research is to develop cellulose sulfate nanofibers (CSNFs) that are not soluble in water and that have a cellulose surface with a high degree of sulfonation. During the sample preparation, the use of chlorosulfonic acid (CSA) can degrade the lignin component and form water-soluble compounds (pulping) and form sulfate groups on the cellulose surface (cellulose sulfonation) [9,10,11,12,13]. The strong charge of the sulfate group on the cellulose surface can introduce repulsive forces between adjacent cellulose fibrils and defibrillate the macroscale fibers into nanoscale fibers or nanofibers. It has been shown that using too much acid could cause increased cleaving of glycosidic linkages in cellulose chains, resulting in a lower degree of polymerization. It has also been shown that the sulfonation reaction of polysaccharides using an ionic liquid medium can dissolve the cellulose chains and form a homogenous reaction [15]. However, in this study, as DMF is a poor solvent for cellulose but a polar protic solvent for CSA, the sulfonation reaction was carried out through a heterogenous substitution with DMF. However, we note that although water is also a polar protic solvent, it cannot be used for sulfonation because CSA can react rapidly with water. It has been reported that sulfonation occurs primarily on the C6 primary alcohol group of cellulose and forms cellulose sulfate, according to spectroscopic analysis [11]. 

### 3.1. Product Yield, Crystallinity, and Morphology of CSNFs

The product yield, crystallinity, and morphology of CSNF products prepared under different reaction conditions were investigated, and the results are shown in Table 1. It is seen that as the amount of CSA increases, the yield decreases. This is expected because CSA is a harsh acid and it can degrade the cellulose component at higher concentrations. In our study, the lowest amount of CSA used of 1.0 mL leads to a product yield of 54.1%. In this case, the molar ratio of cellulose (anhydroglucose) to CSA is 1:1.2, where CSA is in a slight excess to cellulose. 

**Figure 1:**
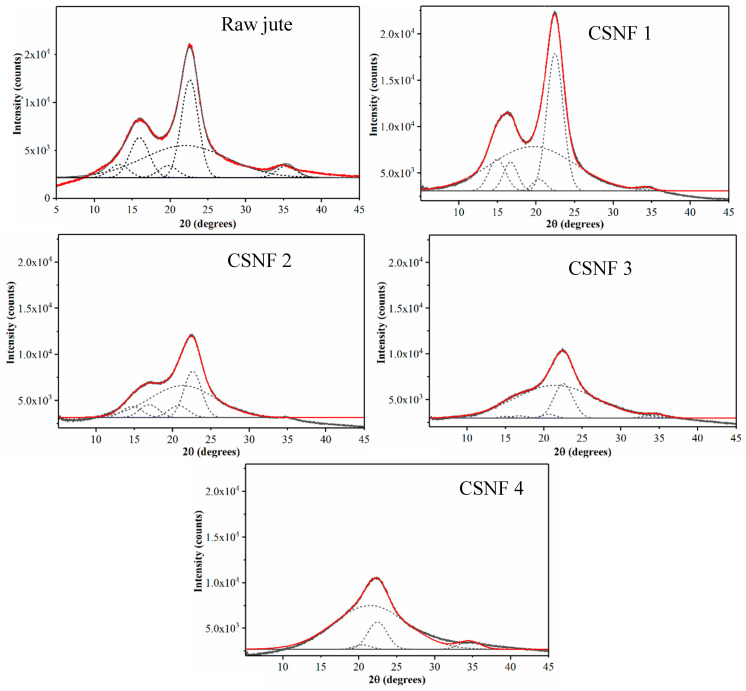
Wide-angle X-ray diffraction (WAXD) patterns of raw jute and cellulose sulfate nanofibers (CSNFs) with peak deconvolutions (dotted lines) to estimate the crystallinity. The red line represents the fit of the experimental data.

At first glance, one may assume that the yield of 54.1% indicates that a significant fraction of biomass components (e.g., lignin and hemicellulose) is removed, leaving behind mostly semi-crystalline cellulose nanofibers. To explore the relationship between the product yield and cellulose crystallinity, WAXD patterns of raw jute and four CSNF samples (as illustrated in Table 1) with peak deconvolution analysis results are illustrated in Figure 1. In this figure, peak areas corresponding to each lattice plane and the amorphous component are determined by the peak deconvolution method, and the results are summarized in Table 2. As shown in this table, the crystallinity of raw jute is around 53.8%, and that of the CSNF 1, CSNF 2, CSNF 3, and CSNF 4 samples is 47.8%, 42.2%, 23.9%, and 19.5%, respectively. The results indicate that as the CSA content increases, the product yield decreases, and the crystallinity decreases. The decrease in product yield exhibits a linear relation, but the decrease in crystallinity shows a non-linear behavior with the CSA content consumed. 

However, upon a closer examination of Figure 1, it becomes apparent that the lattice planes corresponding to 101 and $10\bar{1}$ are depleted with the higher amount of CSA used (these peaks are virtually non-existent in the CSNF 4 sample). In native cellulose I_β_ structure, the $10\bar{1}$ and 002 lattice planes are described as hydrophilic and hydrophobic planes, respectively [16]. This description loosely describes the hydrophilic plane as having exposed hydroxyl groups, while the hydrophobic plane as having buried hydroxyl groups. However, it is generally agreed that both can contribute to the combination of hydrophilic and hydrophobic surfaces, with differences being only marginal [17]. We speculate that because DMF is a hydrophilic solvent, it may be less accessible to the 002 plane and as a result degrade the 002 plane more slowly relative to the other hydrophilic crystalline planes (e.g., $10\bar{1}$). However, this hypothesis needs further verification. Another explanation is that the relative peak areas between 101, $10\bar{1}$, and 002 are similar to those found in raw jute, but as the relative intensity of the amorphous peak increases, deconvolution of the 101 and $10\bar{1}$ planes may become unreliable. As for why the 18.3% decrease in crystallinity occurs in CSNF 3 (when compared to raw jute), it can be attributed to the fact that the molar ratio of cellulose (anhydroglucose) to CSA in CSNF 3 is 1:2.4, where CSA is in greater excess to cellulose. As CSA sulfonates all the accessible primary alcohols on the cellulose surface, it may then start to degrade the cellulose component. 

**Figure 2:**
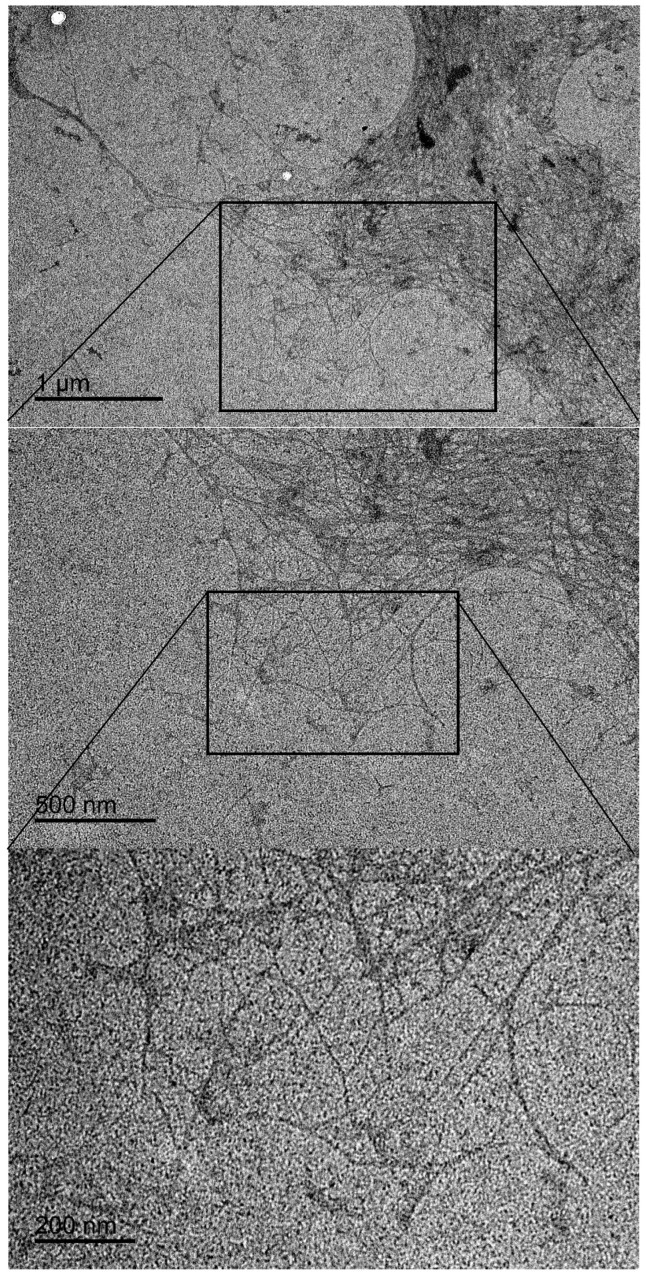
TEM images of CSNF 1 sample at different magnifications.

Figure 2 displays TEM images of CSNF 1 at different magnifications. Nanoscale fibers can be observed, although they are not well dispersed, an indication of only partial fibrillation. There is also presence of dark blob-shaped masses mixed with the fibrillar structure. Considering there is no presence of free salts in the cellulose sulfate after dialysis, this may be concluded to be some organic high-molecular-weight material, such as lignin aggregation. It should be noted that TEM images could only be captured for CSNF 1, indicating that the fibrillar structure in other reactions must be more degraded.

### 3.2. Structure and Composition Characterization of Cellulose Sulfate Nanofibers

**Figure 3:**
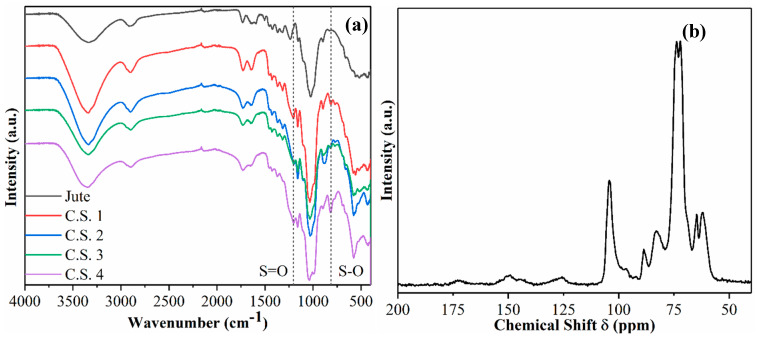
(**a**) Fourier transform infrared spectroscopy (FTIR) of various CSNF samples, and (**b**) ^13^C cross polarization magic-angle spinning nuclear magnetic resonance (CPMAS-NMR) of CSNF 1.

The FTIR spectra of raw jute and various CSNF samples are shown in Figure 3a. Typical bonds associated with cellulose or cellulose derivatives are seen. For example, O-H stretching at 3330 cm^−1^ from the hydroxyl group, C-H stretching at 2900 cm^−1^, and C-O stretching at 1035 cm^−1^ from the glycosidic linkage are seen [18]. Additionally, stretches corresponding to the sulfate ester group are present in the CSNF samples: the S=O stretch is detected at 1204 cm^−1^ and S-O stretch detected at 813 cm^−1^ [13].

The ^13^C CPMAS-NMR spectrum of sample CSNF 1 is shown in Figure 3b, which also exhibits expected peaks typically associated with cellulose and cellulose derivatives. For example, peaks in the region of 63–65 ppm represent the C6 primary alcohol; peaks in the region of 72–75 ppm represent C5, C3, and C2 carbons; peaks in the region of 84–89 ppm represent the C4 carbon; and the peak at 105 ppm represents the C1 carbon. From the NMR spectrum, there is also a very clear indication of the presence of hemicelluloses and lignin. For example, the peak found at 125 ppm is associated with the H-substituted aromatic carbon, typically found on the lignin component [19]. The peak at 150 ppm can be ascribed to C3 and C4 carbons in the guaiacyl units or C3 and C5 carbons in the syringyl units. The peak at 175 ppm is ascribed to carbonyl carbon found in the acetate group of xylan.

**Figure 4:**
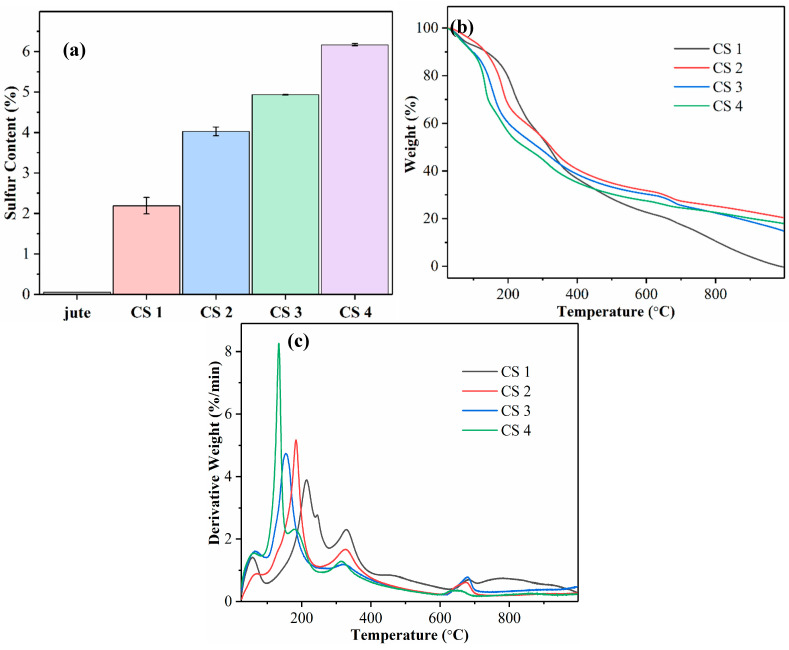
(**a**) Elemental sulfur analysis of raw jute and CSNF samples. (**b**) Thermogravimetric analysis (TGA) and (**c**) derivative thermogravimetry (DTG) curves of CSNF samples.

The elemental sulfur contents of CSNF samples prepared under various sulfonation reactions are shown in Figure 4a. The figure displays an increasing percentage of elemental sulfur detected in reactions using more CSA. Reaction series of the CSNF 1, CSNF 2, CSNF 3, and CSNF 4 samples have elemental sulfur contents of 2.2, 4.0, 4.9, and 6.2 wt%, respectively. There is a slightly greater disparity in sulfur content between the CSNF 1 and CSNF 2 samples, in what otherwise is a linear-like increase in elemental sulfur. It is noted that the increase in sulfur content follows an expected trend, which is also consistent with the notion that the increase in CSA can produce samples with increasing ammonium adsorption capacity that will be discussed later. The elemental sulfur analysis clearly corroborates the presence of sulfonic acid functional groups in the CSNF samples.

The TGA and DTG analysis results of the CSNF samples are shown in Figure 4b,c, respectively. These profiles display a significant lowering of the onset temperatures for degradation in CSNFs as compared to the TGA and DTG profiles of raw jute [2]. In these figures, the most intense derivative weight peak shifts to a lower degradation temperature as the elemental sulfur content increases. It is assumed that the change does not belong to the degradation of lignin, which is known to be around 300–400 °C [20]. Because the degree of sulfonation on the cellulose surface is systematically varied, it is conceivable that the observed shift in degradation temperature is caused by an increasingly substituted cellulose derivative. As such, a possible explanation is acid-catalyzed dehydration of the alcohol group in cellulose. Research on the effects of acid-impregnated cellulose on pyrolysis has shown a decrease in the degradation temperature [21]. The peak at 330 °C is present for all samples at the same temperature and is ascribed to lignin. The peak at 680 °C is ascribed to the degradation of the high-molecular-weight lignin component. 

### 3.3. Evaluation of Ammonium Removal Efficiency

**Figure 5:**
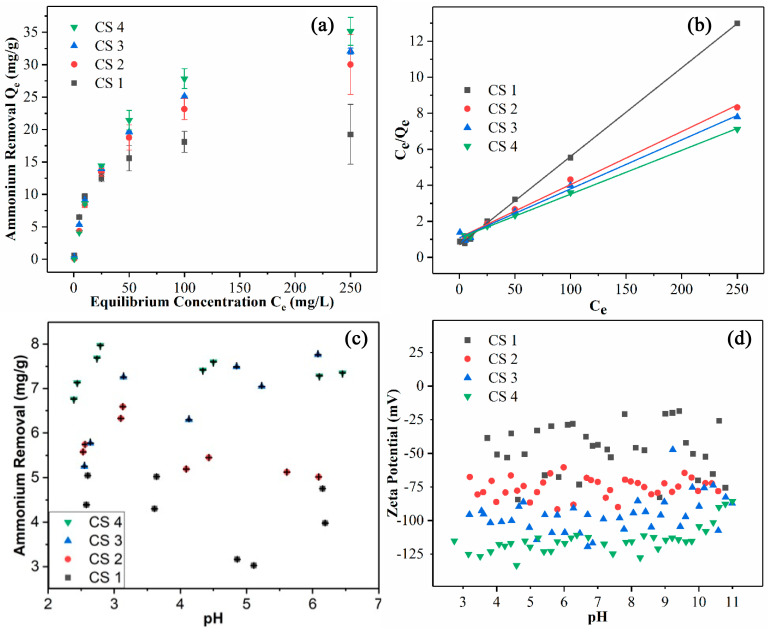
(**a**) The equilibrium adsorption capacity of CSNFs as a function of the equilibrium ammonium concentration. (**b**) Langmuir isotherm fittings for various CSNF samples. (**c**) Ammonium adsorption (removal) capacities of CSNFs as a function of the pH value. (**d**) Zeta potential measurements of CSNFs at various pH levels.

To evaluate the ammonium removal efficiency of the CSNFs, the following analyses were carried out. Firstly, the equilibrium adsorption capacity for ammonium removal (*Q_e_*) was plotted as a function of the equilibrium ammonium concentration (*C_e_*) for various CSNF samples (Figure 5a). In this figure, all adsorption curves follow an expected trend, i.e., the adsorption capacity increases rapidly initially, and then the increase tapers off, reaching an asymptote. The plateau values of *Q_e_* for CSNF 1, CSNF 2, CSNF 3, and CSNF 4 are 19.3, 30.0, 32.1, and 35.1 mg of ammonium per gram of CSNF, respectively. A higher adsorption capacity is observed in the CSNFs prepared with a greater amount of CSA.

The ammonium removal results in Figure 5a were analyzed using the Langmuir isotherm model, which assumes that the adsorption process follows the monolayer mechanism. In this analysis, the *C_e_/Q_e_* value was plotted against *C_e_* (Figure 5b). The figure indicates that the data exhibit a linear relationship for each CSNF sample. The fitting results are summarized in Table 3, showing that all fits have a R^2^ value larger than 0.99. These results support the use of the Langmuir isotherm model. The isotherm data were also analyzed with the Freundlich isotherm model, assuming that the adsorption follows the multilayer deposition pathway. But this analysis yielded a significantly lower R^2^ value, indicating that the ammonium adsorption by the CSNFs follows the monolayer process.

Using Equation (1), the maximum adsorption capacity (*Q_m_*) can be calculated as the inverse of the slope in Figure 5b. All the *Q_m_* values for the CSNFs (CSNF 1: 20.3 mg/g, CSNF 2: 34.0 mg/g, CSNF 3: 36.8 mg/g, CSNF 4: 41.1 mg/g) are higher than those of carboxylated cellulose nanofibers produced by the nitro-oxidization or TEMPO-mediated oxidation approach (*Q_m_* = 22.7 mg/g for nitro-oxidized CNF, and *Q_m_* = 18.2 mg/g for TEMPO-oxidized CNF) [22]. However, we note that although the sulfonated nanocellulose system exhibits better ammonium adsorption performance than the carboxylated nanocellulose system, the current sulfonation approach involves the use of DMF that requires the recycling of spent waste. We further note that the *Q_m_* value of the best performing CSNF 4 sample (41.1 mg/g) is significantly better than that of zeolite (6.3 mg/g) [23], mordenite (9.5 mg/g) [24], clinoptilolite (11.2 mg/g) [25], and biochar (2.8 mg/g) [26] in the literature, and it is approaching the best reported *Q_m_* value of a synthetic polymeric hydrogel (42.7 mg/g) [27].

The effects of pH level on the adsorption capacity and zeta potential of the CSNF samples were also investigated, and the results are illustrated in Figure 5c,d, respectively. Figure 5c displays the effective ammonium removal capacity in the pH range of 2.5–6.5. This pH range was chosen to ensure that the ammonium would not be converted to ammonia, since ammonium, with a pKa of 9.3, can convert to ammonia at higher pH values. Based on the Henderson–Hasselbalch equation, it can be calculated that at pH = 7.3, there is a 1% conversion of ammonium to ammonia. While ammonia is soluble in water, it exists as a gas and can evaporate. To avoid these issues, the chosen pH value of the adsorption study was below 7.0. In Figure 5c, it is seen that the adsorption capacity of the CSNFs generally increases with the content of CSA used. In fact, a higher ammonium adsorption capacity can be correlated with the increase in negative zeta potential of the sample (Figure 5d). These results confirm that that the surface charge due to the sulfonic acid group of the CSNFs cannot be effectively screened with the increase in acid, while the sulfonic acid groups remain highly selective for adsorbing higher-molecular-weight cations, such as ammonium ions. The zeta potentials of the various CSNF samples were investigated with a wider pH range between 3 and 11 (Figure 5d). It is seen that the zeta potentials remain about constant in each sample, which can be attributed to the strong acidic characteristics of the sulfonic acid group, i.e., it is not protonated under the chosen acidic conditions, unlike carboxylic acid. The above properties of the CSNFs are in contrast to those of carboxylated nanocellulose, whose zeta potential and ammonium adsorption capacity both approach 0 as the acidity is increased. This study indicates that cellulose sulfate nanofibers can be used in a more robust manner than carboxylated cellulose nanofibers.

## 4. Conclusions

Sulfonation of raw lignocellulosic biomass can create cellulose sulfate nanofibers directly, where the presence of sulfonic acid groups on the nanofiber surface are more effective to adsorb ammonium cations in a relatively broad pH range (~2.5–neutral) than carboxylated cellulose nanofibers. In this study, the sulfonation approach involved the use of chlorosulfonic acid and N,N-dimethylformamide. An increase in chlorosulfonic acid content can severely decrease the yield and crystallinity of cellulose sulfate nanofibers. The presence of nanofibers was confirmed when using a nearly stoichiometric quantity of chlorosulfonic acid relative to anhydroglucose units in the cellulose. The structure, composition, and functionality of the cellulose sulfate nanofibers were characterized by Fourier transform infrared spectroscopy, nuclear magnetic resonance spectroscopy, and elemental sulfur and thermogravimetric analysis techniques. The sulfonation reaction appears to occur first on lignin and hemicelluloses, but the pulping mechanism remains unclear. The best performing cellulose sulfate nanofiber sample exhibits the maximum ammonium adsorption capacity of 41.1 mg/g, which approaches the performance of the best synthetic adsorbents. Both the ammonium adsorption capacity and zeta potential values of the cellulose sulfate nanofibers remain about constant at low pH values (as low as 2.5). Although the cellulose sulfate nanofibers exhibit more robust ammonium adsorption performance than carboxylated nanofibers, a more facile and sustainable method to simultaneously pulp the lignocellulosic biomass and create sulfonic acid groups on the resulting nanocellulose surface needs to be further explored.

## Figures and Tables

**Table 1:** Reaction conditions for preparation of various CSNF samples.

CSNF	Raw Jute (g)	DMF (mL)	Time (Hours)	CSA (mL)	Yield (%)
1	2.0	35	6	1.0	54.1
2	2.0	35	6	1.5	50.3
3	2.0	35	6	2.0	47.5
4	2.0	35	6	2.5	44.2

**Table 2:** Peak deconvolution results of WAXD patterns of raw jute and CSNF samples in Table 1.

Raw Jute				
Peak (Index)	Area	FWHM	Peak position	Area (%)
1 (101)	4,313.1	3.0	13.2	4.3
$2\;(10\bar{1}$)	13,371.7	3.0	15.9	13.2
3 (amorphous)	46,812.4	13.2	22.0	46.2
4 (021)	3,958.2	3.0	19.7	3.9
5 (002)	28,842.5	2.6	22.6	28.5
6 (040)	3981.1	3.0	35.4	3.9
**CSNF 1**				
Peak (Index)	Area	FWHM	Peak position	Area (%)
1 (101)	9,317.6	2.6	15.0	7.5
$2\;(10\bar{1}$)	7,116.5	2.2	16.7	5.7
3 (amorphous)	65,030.8	12.8	19.6	52.2
4 (021)	2,718.1	2.0	20.4	2.2
5 (002)	39,594.5	2.5	22.5	31.8
6 (040)	717.9	2.0	34.3	0.6
**CSNF 2**				
Peak (Index)	Area	FWHM	Peak position	Area (%)
1 (101)	4,004.2	3.1	14.7	6.2
$2\;(10\bar{1}$)	4,463.5	2.9	16.9	6.9
3 (amorphous)	37,201.5	10.0	21.3	57.8
4 (021)	4,207.8	3.0	20.8	6.5
5 (002)	14,452.1	2.7	22.6	22.5
6 (040)	~0	2.0	34.0	~0
**CSNF 3**				
Peak (Index)	Area	FWHM	Peak position	Area (%)
1 (101)	452.9	2.0	15.0	0.7
$2\;(10\bar{1}$)	720.1	2.6	16.8	1.1
3 (amorphous)	47,997.6	12.5	21.5	76.1
4 (021)	1,021.1	2.2	20.6	1.6
5 (002)	11,760.9	2.9	22.5	18.6
6 (040)	1,155.0	3.3	34.4	1.8
**CSNF 4**				
Peak (Index)	Area	FWHM	Peak position	Area (%)
1 (101)	~0	2.6	14.4	~0
$2\;(10\bar{1}$)	~0	2.6	16.7	~0
3 (amorphous)	4,802.1	11.2	21.5	80.5
4 (021)	482.9	2.6	20.5	1.9
5 (002)	3,000.9	3.0	22.5	13.4
6 (040)	821.9	3.4	34.6	4.2

**Table 3:** Langmuir isotherm fitting parameters for cellulose sulfate (CS).

CSNF	Intercept	Slope	R^2^	*Q_m_* (mg/g)
1	0.676	0.0492	0.999	20.329
2	1.096	0.0294	0.996	33.990
3	1.079	0.0272	0.992	36.791
4	1.071	0.0243	0.998	41.084

## Data Availability

Data are contained within the article.

## References

[1] Isogai A., Saito T., Fukuzumi H. (2011). TEMPO-oxidized cellulose nanofibers. Nanoscale.

[2] Sharma P.R., Joshi R., Sharma S.K., Hsiao B.S. (2017). A simple approach to prepare carboxycellulose nanofibers from untreated biomass. Biomacromolecules.

[3] Jensen D., Weiss J., Rey M.A., Pohl C.A. (1993). Novel weak acid cation-exchange column. J. Chromatogr. A.

[4] Kunin R., Vassiliou B. (1964). New deionization techniques based upon weak electrolyte ion exchange resins. Ind. Eng. Chem. Process Des. Dev..

[5] Chen Y.G., Sofińska-Chmiel W., Lv G.Y., Kołodyńska D., Chen S.H. (2021). Application of modern research methods for the physicochemical characterization of ion exchangers. Materials.

[6] Basu S., Debnath A.K., Basu S., Debnath A.K. (2015). Chapter II—Main Equipment. Power Plant Instrumentation and Control Handbook.

[7] Davis J.R., Chen Y., Baygents J.C., Farrell J. (2015). Production of acids and bases for ion exchange regeneration from dilute salt solutions using bipolar membrane electrodialysis. ACSNF Sustain. Chem. Eng..

[8] Meyers P., Gottlieb L., DeSilva F. (2013). Lead removal by ion exchange. Recycling of Metals and Engineercd Materials.

[9] Luo J., Semenikhin N., Chang H., Moon R.J., Kumar S. (2018). Post-sulfonation of cellulose nanofibrils with a one-step reaction to improve dispersibility. Carbohydr. Polym..

[10] Mestechkina N.M., Shcherbukhin V.D. (2010). Sulfated polysaccharides and their anticoagulant activity: A review. Appl. Biochem. Microbiol..

[11] Zhang K., Brendler E., Geissler A., Fischer S. (2011). Synthesis and spectroscopic analysis of cellulose sulfates with regulable total degrees of substitution and sulfation patterns via ^13^C NMR and FT Raman spectroscopy. Polymer.

[12] Strätz J., Liedmann A., Trutschel M.-L., Mäder K., Groth T., Fischer S. (2019). Development of hydrogels based on oxidized cellulose sulfates and carboxymethyl chitosan. Cellulose.

[13] Pingrey B., Hsieh Y.-L. (2022). Sulfated cellulose nanofibrils from chlorosulfonic acid treatment and their wet spinning into high-strength fibers. Biomacromolecules.

[14] Chen X. (2015). Modeling of experimental adsorption isotherm data. Information.

[15] Liu X., Chen T., Hu Y., Li K., Yan L. (2014). Catalytic synthesis and antioxidant activity of sulfated polysaccharide from *Momordica charantia* L.. Biopolymers.

[16] Wohlert M., Benselfelt T., Wågberg L., Furó I., Berglund L.A., Wohlert J. (2022). Cellulose and the role of hydrogen bonds: Not in charge of everything. Cellulose.

[17] Malaspina D.C., Faraudo J. (2019). Molecular insight into the wetting behavior and amphiphilic character of cellulose nanocrystals. Adv. Colloid Interface Sci..

[18] Kačuráková M., Capek P., Sasinková V., Wellner N., Ebringerová A. (2000). FT-IR study of plant cell wall model compounds: Pectic polysaccharides and hemicelluloses. Carbohydr. Polym..

[19] Vane C.H., Drage T.C., Snape C.E., Stephenson M.H., Foster C. (2005). Decay of cultivated apricot wood (*Prunus armeniaca*) by the ascomycete *Hypocrea sulphurea*, using solid state 13C NMR and off-line TMAH thermochemolysis with GC–MS. Int. Biodeterior. Biodegrad..

[20] Brebu M., Vasile C. (2010). Thermal degradation of lignin—A review. Cellul. Chem. Technol..

[21] Long Y., Yu Y., Chua Y.W., Wu H. (2017). Acid-catalysed cellulose pyrolysis at low temperatures. Fuel.

[22] Johnson K.I., Ilacas G., Das R., Chang H.Y., Sharma P.R., Dimkpa C.O., Hsiao B.S. (2024). A circular solution to enhance the food-water nexus by nanocellulose technologies for ammonium recovery and reuse. Sustain. Sci. Technol..

[23] Widiastuti N., Wu H., Ang H.M., Zhang D. (2011). Removal of ammonium from greywater using natural zeolite. Desalination.

[24] Weatherley L.R., Miladinovic N.D. (2004). Comparison of the ion exchange uptake of ammonium ion onto New Zealand clinoptilolite and mordenite. Water Res..

[25] Wang Y., Liu S., Xu Z., Han T., Chuan S., Zhu T. (2006). Ammonia removal from leachate solution using natural Chinese clinoptilolite. J. Hazard. Mater..

[26] Vu N.-T., Do K.-U. (2021). Insights into adsorption of ammonium by biochar derived from low temperature pyrolysis of coffee husk. Biomass Convers. Biorefinery.

[27] Zheng Y., Liu Y., Wang A. (2011). Fast removal of ammonium ion using a hydrogel optimized with response surface methodology. Chem. Eng. J..

